# On the Role and Effects of Uncertainties in Cardiovascular *in silico* Analyses

**DOI:** 10.3389/fmedt.2021.748908

**Published:** 2021-12-01

**Authors:** Simona Celi, Emanuele Vignali, Katia Capellini, Emanuele Gasparotti

**Affiliations:** ^1^BioCardioLab, UOC Bioingegneria, Fondazione Toscana Gabriele Monasterio, Massa, Italy; ^2^Department of Information Engineering, University of Pisa, Pisa, Italy

**Keywords:** uncertainty quantification, aorta, computational methods, CFD - computational fluid dynamics, FSI - fluid structure interaction, RBF - radial basis functions

## Abstract

The assessment of cardiovascular hemodynamics with computational techniques is establishing its fundamental contribution within the world of modern clinics. Great research interest was focused on the aortic vessel. The study of aortic flow, pressure, and stresses is at the basis of the understanding of complex pathologies such as aneurysms. Nevertheless, the computational approaches are still affected by sources of errors and uncertainties. These phenomena occur at different levels of the computational analysis, and they also strongly depend on the type of approach adopted. With the current study, the effect of error sources was characterized for an aortic case. In particular, the geometry of a patient-specific aorta structure was segmented at different phases of a cardiac cycle to be adopted in a computational analysis. Different levels of surface smoothing were imposed to define their influence on the numerical results. After this, three different simulation methods were imposed on the same geometry: a rigid wall computational fluid dynamics (CFD), a moving-wall CFD based on radial basis functions (RBF) CFD, and a fluid-structure interaction (FSI) simulation. The differences of the implemented methods were defined in terms of wall shear stress (WSS) analysis. In particular, for all the cases reported, the systolic WSS and the time-averaged WSS (TAWSS) were defined.

## 1. Introduction

The world of cardiovascular simulations for the evaluation of implants and devices is acquiring more importance. The numerical approaches constitute a new valuable resource for clinical design and their accuracy is a fundamental requirement. Nevertheless, several issues are still present due to the interaction between the device and the surrounding biological tissues. Indeed, *in silico* models require the setting of several parameters that are usually affected by uncertainties as a consequence of either measurement errors and/or natural physiological variability. These uncertainties involve different levels: (i) imaging (structural and functional), (ii) segmentation, (iii) material characterization/modeling and (iv) computational model.

*Imaging* - In recent years, imaging has acquired increasing relevance for investigation potential in terms of both structural and functional information. Three-dimensional morphology reconstruction has improved due to the advances of the last generation of technologies. CT, MRI, and echographic imaging (ECHO) techniques are currently used to acquire high-resolution dynamic images. In the context of cardiovascular images for aortic analysis, these data play a fundamental role in computational image-based modeling of patient-specific cases.

A faithful reconstruction of cardiovascular structures is at the basis of computational modeling. Morphological information can be acquired mainly by adopting a segmentation algorithm of CT and MRI data. ECHO techniques might also be adopted but their applications are usually limited to diagnostic parameters assessment rather than computational domains definition. In comparison with other modes, CT is the technique with the best resolution available (1, 2): its standard spatial resolution is usually around 0.5 mm, but it can reach smaller values (3) according to the last generation scanners. Given these features, literature studies report a significant contribution of CT-based techniques for aortic structures assessment (4, 5). The improved spatial resolution is at the basis of cardiovascular morphological definition (6) and patient-specific reconstruction of computational domains (7) for biomechanical and fluid dynamic analysis. Despite the high level of resolution, Parodi et al. (8) individuated the possible sources of error in aortic diameter estimation *via* CT measurement.

The MRI methods exhibit the same multiplanar imaging capabilities of CT, however, they present lower spatial resolution. Structural information from MRI data can reach a maximum resolution of few milliliters (9), causing the insurgence of possible partial volume effect. Nevertheless, the potential of MRI lies in its versatility, as new acquisition procedures are continuously developed. For example, T1-weighted ECG-gated acquisitions for the measurement of aortic wall thickness were reported in the literature (10). This aspect has a pivotal role in the numerical analysis of aortic biomechanics, given the fact that usually assumptions are required to obtain a full reconstruction including the wall thickness.

As well as for structural modalities, functional imaging provides useful information for the numerical hemodynamic simulations in terms of blood flow assessment in the aorta. ECHO is the reference standard for two-dimensional blood flow velocity analysis. However, its main limitations are given by 2-D acquisitions and velocity encoding confined to a single direction, determined by the probe position (11). The assessment of complex three-dimensional hemodynamics holds added value for computational simulations, both in terms of numerical modeling and validation. Four-dimensional flow MRI (4D flow) sequences, such as specific phase contrast (PC-MRI), enable qualitative and quantitative analysis of blood flows in different districts, including the heart and great arteries (12). PC-MRI procedures allow for qualitative and quantitative assessment of blood velocity during the cardiac cycle. In recent years, the contribution of the PC-MRI technique was highlighted in the context of numerical hemodynamic simulations, especially for aortic and aneurysmatic structures, as a tool for the definition of patient-tailored inlet conditions (13) and flow patterns validation (14). State of the art assessment of 4D flow uncertainty was carried out on the aorta and the carotid bifurcation (15). The study adopted a Monte Carlo method to propagate the noise at the local level up to the global image. The results produced flow uncertainty maps, mainly linked to image noise. The technique was demonstrated to be feasible for the flow pattern quantification inside the whole aortic complex, with satisfactory levels of signal-to-noise ratio (SNR). Nevertheless, the same level of MRI data inaccuracies already discussed in the previous subsection remains. In fact, it is worth underlining that MRI's low spatial resolution prevents the correct estimation of the calculation of parameters such as wall shear stress (WSS) and oscillatory shear index (OSI) (16). Some studies relied on the calculation of WSS parameters from 4D flow data (17, 18), but computational tools remain the most reliable approach. For this reason, PC-MRI data are used to obtain reliable patient-specific flow inlet conditions in numerical computations rather than direct WSS estimations. However, it is necessary to stress out that uncertainties originating from functional MRI data acquisition and processing can also propagate at the simulated output (19). In literature, the effect of the uncertainties of inlet conditions from PC-MRI processing and of their propagation was analyzed in the particular context of the aorta. Bozzi et al. (20) contributed to the assessment of PC-MRI profile inaccuracies by using a Monte Carlo simulation set. A significant influence of WSS and blood pressure emerged. It was demonstrated that the boundary condition modeling strategy also affects the fluid dynamic results: the choice of 1D or 3D flow profiles from PC-MRI processing produced non-negligible differences in the simulation outcomes.

*Segmentation* - It is worth underlining that the reconstruction results are strongly dependent on the imaging technique adopted and operator dependant as well (21). Even though semi-automatic segmentation algorithms are available (22), the operator influence in geometry reconstruction remains. Different segmentation tools, including both manual and machine-learning-based produce valid outcomes. However, the segmentation results might not be absolute and reproducible, given the huge variety of availble tools and the lack of a standardized method. The last reported trend on image segmentation concerns the adoption of deep learning methods. The deep learning techniques offer significant benefits in terms of speed and performance (23, 24). Still, error propagation requires precise assessment. State of the art characterizations of these methods were mainly focused on aortic structure segmentations to obtain fractional flow reserve simulations. Maher et al. (25), in a more recent study, analyzed the performance metrics of automatic neural-network-based segmentation. The segmented structures included cerebral, pulmonary arteries, and ascending/descending aorta portions, with both 2D and 3D clinical CT/MRI datasets. The study group concluded that the inaccuracies and uncertainties produced by the automated process were in line with the results from manual segmentation carried out by expert clinicians.

*Material modeling* - Material modeling also remains a significant source of inaccuracies. Cardiovascular tissue characterization is limited by *ex vivo* tissue availability. However, different *in vivo* techniques for the assessment of mechanical properties of cardiovascular tissues are reported, with particular attention on the aortic district. The current state of the art presents different proofs of concept concerning these approaches, which are mainly based on CT/MRI data processing (26–30). To achieve a complete material assessment, aortic mechanical characterization is made possible by tissue harvesting from valve replacement procedures. The state of the art presents a wide range of studies of tissue mechanical assessments relying on biaxial traction tests (31–33). Nevertheless, uncertainties remain present at different levels: (i) patient physiological variability affects the data evaluation (34); (ii) evaluation methods are not always reliable, as different groups still adopt uniaxial tensile tests (35), and (iii) constitutive modeling still requires an uncertainty quantification to assess the effects on the simulation output. This last aspect is particularly true for fiber-based anisotropic material models, which present an elevated number of constitutive parameters and might carry a significant level of required accuracy. Error analyses provided by the state of the art confirm the performances of the hyperelastic anisotropic models (36, 37). Nevertheless, it is worth underlining that, even if the fiber-based models are suitable to cope with the hyperelastic and anisotropic nature of the aortic tissue, different groups still adopt linearized approaches to model the tissue behavior in numerical approaches, also to reduce the model complexity, and lighten the computational load (38–40). The linearization approach can be justified by the assumption of small deformations occurring between the systolic and diastolic phases in the cardiac cycle.

*Computational model* - Another source of uncertainty for the analysis of aortic stress and hemodynamics arises from the notion that the computational model is an approximation of real physics, due to the intrinsic complexity of the phenomenon. In this context, a technique to be used as a gold standard is still lacking and different approaches are presented in the state of the art. In literature, structural and hemodynamic studies are presented. In the first case, the blood pressure is imposed as a boundary condition (41, 42), while in the second case the pressure load derives directly from the hemodynamics. For the fluid dynamic approach, computational fluid dynamics (CFD) (43, 44) or fluid-structure interaction (FSI) (45, 46) are commonly used. FSI accounts for multiphysics phenomena and it is, therefore, preferable in comparison with CFD. Nevertheless, it remains a very complex task to handle, and it is computationally expensive. Additionally, the FSI approach only partially accounts for the vessel movement caused by the heart contraction. To overcome the FSI limitations and to reduce the computation weight, more recently, CFD based on morphing with radial basis functions (RBF) (47–49) were proposed. Another point of discussion is given by the boundary conditions definition. A resourceful tool for this purpose is given by functional clinical images and relative processing. Contrast phase MRI images were reported to be analyzed for the definition of inlet fluid dynamic conditions at the aortic valve level for evaluation of the aorta. The added value of this technique is the introduction of patient-specific conditions even at the fluid dynamic level. Nevertheless, it was demonstrated that inaccuracies in the stroke volume and heart rate estimation propagate significant errors in numerical simulations (38). The inaccuracy effects are particularly evident at the systolic peak level and in the early diastole. On the other hand, the effect of flow distribution at the aortic valve level is negligible as long as no valve pathologies are involved (50–52).

The aim of the current study is to evaluate the effects of uncertainties on aortic computational modeling at different levels. First, the effects of segmentation and surface smoothing were defined, then, three different simulation methods were setup for the geometry under analysis. Finally, the results in terms of WSS and time averaged WSS (TAWSS) assessment are presented and discussed.

## 2. Methods

**Figure 1:**
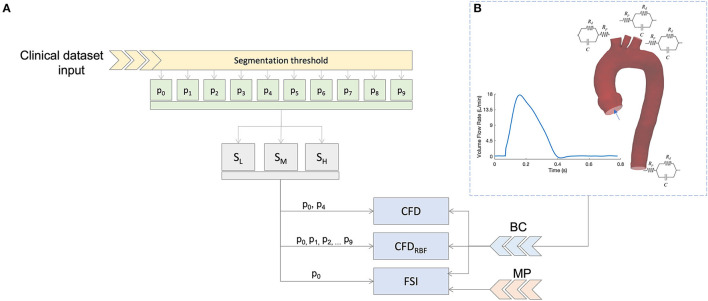
Summary of the simulation scheme **(A)** and boundary condition **(B)** based on 3D-0D coupling with three element Windkessel models (3WKM) model. The same boundary conditions (BC) was applied for all models, specific material properties (MP) were used for the fluid-structure interaction (FSI) simulation.

In this section, the workflow and the decision tree adopted to compare the fluid dynamic results are defined, as depicted in Figure 1. The workflow includes three main choices for the modeling of the aorta:

1. the phase (*p*_*n*_) of the cardiac cycle used for the segmentation;
2. the level of surface smoothing to adopt: low, medium, or high (*S*_*L*_, *S*_*M*_,*S*_*H*_);
3. the numerical approach to use.

In the following subsections, each of these points will be described.

### 2.1. Image Processing - Segmentation

The tomographic dataset was acquired with a 320-detector scanner (Toshiba Aquilon One, Toshiba, Japan). The ECG-gated cardiac CT scans were performed in one cardiac cycle, a total of 10 ECG-gated phases were acquired. The chosen phases were taken within the ECG R-R interval with a fixed time step of 78 ms. In this way, it was possible to sample the aortic phases with a 10% resolution. The segmentation process was performed with the functions provided by the VMTK package (Vascular Modeling Toolkit, www.vmtk.org). In particular, a threshold algorithm with the same threshold level was imposed for all the phases. Three-dimensional geometrical models of ascending aorta, arch, and supra-aortic vessels were generated for each cardiac phase. The segmented models were exported as stereolithography (STL) file format for accomplishing the numerical simulation setup.

### 2.2. Image Processing - Surface Smoothing

**Figure 2:**
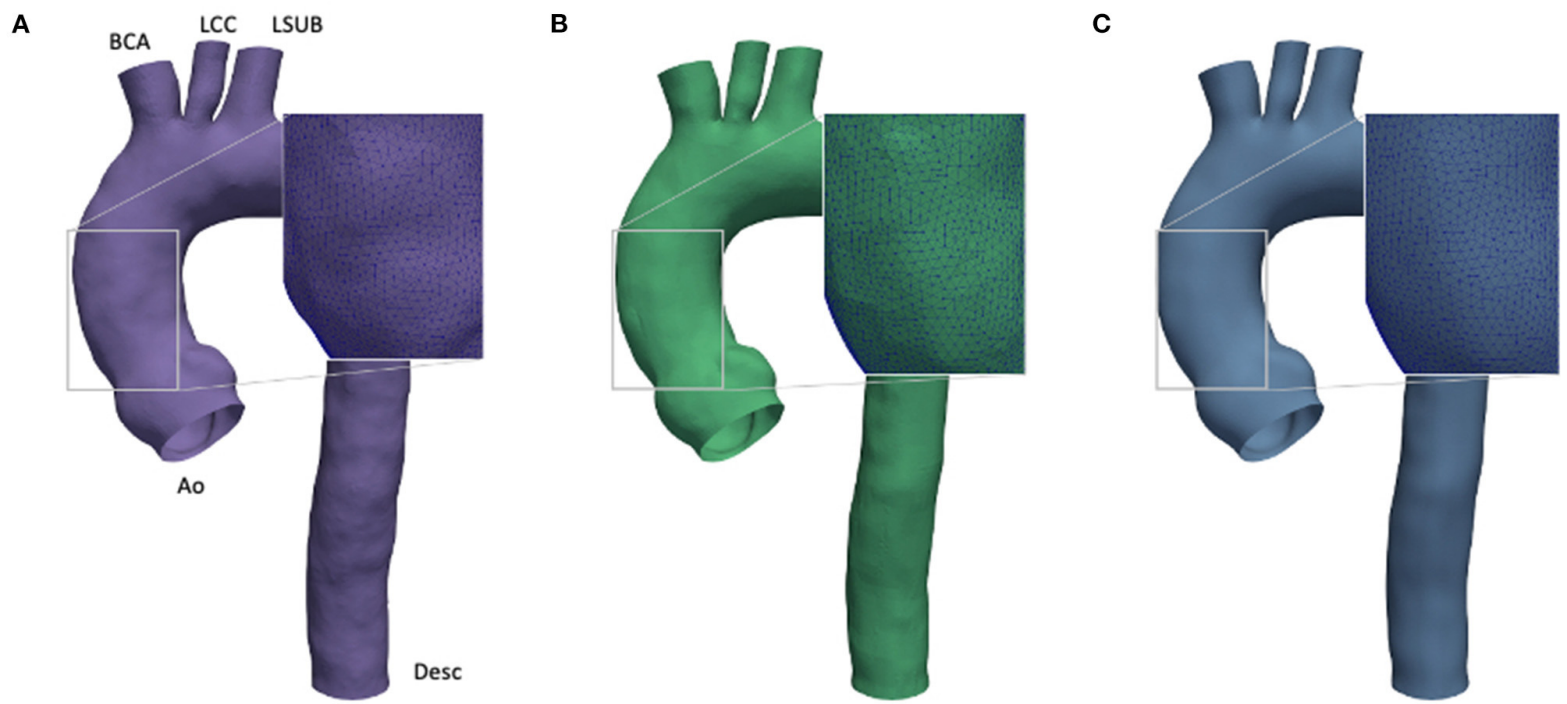
Comparison of effect of different smoothing levels. Segmented aortic geometry at phase *p*_0_ with smoothing: low (*S*_*L*_) **(A)**, medium (*S*_*M*_) **(B)**, and high (*S*_*H*_) **(C)**.

**Figure 3:**
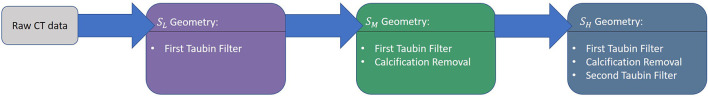
Schematic of the cumulative smoothing strategy adopted for the aortic morphology.

In numerical simulations workflows, the boundaries of the fluid geometry are modeled as smooth, however, the raw geometry obtained directly from medical imaging does not produce the required level of smoothness, and hence, it has to be pre-processed. The main cause is given by low resolution and artifacts, as already discussed in the previous section. Currently, different strategies are available to achieve smooth 3D patient-specific reconstructions. Given this wide variety of approaches, it is difficult to assess if the filtering process causes uncertainties. To account for this source of errors, a preliminary investigation was performed on a geometry with three different levels of smoothness (*S*_*L*_, *S*_*M*_, and *S*_*H*_) as depicted in Figure 2. This investigation was set to define the most suitable smoothing level to use for the computational models. In particular, this process was carried out by applying a shape-preserving Taubin smoothing filter with a weighting factor (*w*) of 0.5 and the maximum number of iterations (*n*) of 15 (53). To achieve different levels of smoothness, the same filter was applied multiple times according to a smoothing strategy summarized in three main steps (refer to Figure 3). Also, the presence of calcifications was considered, given their expected effect on the numerical results (54):

1. Raw DICOM → *S*_*L*_: first application of global Taubin filter (*w* = 0.5, *n* = 15);
2. *S*_*L*_ → *S*_*M*_: local removal of calcification artifacts;
3. *S*_*M*_ → *S*_*H*_: second application of global Taubin filter (*w* = 0.5, *n* = 15);

The Taubin filter (55) was applied by using the implemented routine from the VMTK package. The presence of calcification artifacts within the ascending aorta region was individuated with the support of a skilled clinician. All the 3D segmented models included the ascending aorta, the aortic arch, the descending aorta (*Desc*), and the apico-aortic branches (brachiocephalic artery (*BCA*), left common carotid artery (*LCC*), and left subclavian artery (*LSUB*), as shown in Figure 2A). The evaluation of the smoothing effect was carried out exclusively on the CFD approach at the *p*_0_ phase.

### 2.3. Governing Equations and Simulations

After defining the clinical images processing method, the different computational techniques adopted in the study are reported. In particular, three different approaches are investigated and compared:

- Computational fluid dynamics;
- Computational fluid dynamics with RBF morphing on the aortic wall (CFD_*RBF*_);
- Fluid-structure interaction;

In this subsection, we describe the common and specific settings for each approach.

For the fluid dynamics calculations, the same computational setup was assumed for all models. The pulsatile flow behaviors were analyzed by solving the 3D incompressible Navier-Stokes equation system:


(1) $$\begin{array}{l} \begin{array}{c} \rho \frac{\partial v}{\partial t}+\rho \left(v\cdot \nabla \right)v-\nabla \sigma =0\\ \nabla \cdot v=0 \end{array} \end{array}$$

where ρ is the density of the fluid, *v* is the blood velocity, and σ is the Cauchy stress. Blood was modelled as an incompressible Newtonian fluid, with constant values of density 1,060 *kg*·*m*^3^ and dynamic viscosity 0.0035 *kg*(*m*·*s*)^−1^. Blood flow was assumed laminar, as the Reynolds number estimated from the worst configuration at the systolic peak was equal to 3,534. The proposed laminar hypothesis is in line with previous literature studies (40, 56–58).

For all the simulations, the fluid domain was partitioned in tetrahedral elements using Ansa (BetaCAE) with four boundary layers of triangular prisms. Ansys Fluent (ANSYS Inc. Canonsburg, PA, USA) was used as the solver. A mesh sensitivity analysis was carried out on the given aortic case. In particular, the mesh size was reduced and the average value of WSS in the ascending aorta region was monitored. The mesh size was chosen after reaching a WSS variation below 0.1%. After the analysis, the models were discretized with a tetrahedral mesh with a mean element size of 1 mm. Prism elements were added to the vessel wall by implementing 4-layers inflation for a total thickness of 1.2 mm and a growth-rate equal to 1.5.

For the CFD simulations, two geometries were considered to assess the variability linked with phase segmentation. In particular, the geometries were taken from two phases of the cardiac cycle: one at diastole (*p*_0_) and one at the systolic peak (*p*_4_) (refer to Figure 1).

For the CFD_*RBF*_, the simulation scheme was implemented according to previous studies (47, 48). Briefly, the method imposes patient-specific aortic wall motion during the cardiac cycle without re-meshing using a morphing approach. It is worth pointing out that for this method all the phases from the segmentation were considered to calculate the radial basis function solution. Additionally, no material property estimation was required, as the method only relies on the knowledge of wall displacement at the different phases.

At last, for the FSI simulation, a fully-coupled partitioned approach was implemented. In particular, the FSI coupling used an Arbitrary Lagrangian-Eulerian method to transmit the wall displacement and the pressure between the fluid and the structural domains of the simulation (59). An isotropic linear elastic material behavior was assumed for the aortic wall, with the hypothesis of small deformations in the cardiac cycle (39). In order to guarantee a comparable wall displacement between the FSI and CT-gated results, the Young modulus (*E*) was estimated according to Laplace's law for the calculation of stresses within a pressurized membrane and the strain estimation from the normalized variation of vessel diameter between systole (*p*_4_) and diastole phase (*p*_0_). The wall thickness (*t*) was set equal to 2 mm (32) and the resulting *E* was equal to 0.5 MPa.

#### 2.3.1. Boundary Conditions

The same boundary conditions were imposed for the CFD, CFD_*RBF*_, and FSI, as also depicted in Figure 1B. In practice, a blood flow velocity inlet profile was assigned as a waveform to the aortic inlet (*Ao*). The values of velocity for the inlet were extracted directly from patient-specific ECG-gated ECHO signals at the aortic site. The corresponding flow profile resulted in a cardiac output of 4.5 l/min with an heart rate of 77 bpm. Three cardiac cycles of the given flow profile were considered to achieve periodic fully developed solutions and to eliminate nonlinear start-up effects. The solution was considered at the last cycle.

Concerning the supra-aortic vessels and the descending aorta, a pressure outlet condition was imposed by coupling the 3D domain with three-elements Windkessel models (3WKM) (38). The 3WKM is a numerical model relying on the circuital-hydraulic analogy to establish a relation between the pressure *P*(*t*) and the flow rate *Q*(*t*) at a given outlet branch. The following partial differential equation is assumed:


(2) $$\begin{array}{l} \left(1+\frac{R_{p}}{R_{d}}\right)Q\left(t\right)+CR_{p}\frac{dQ\left(t\right)}{dt}=C\frac{dP\left(t\right)}{dt}+\frac{P\left(t\right)}{R_{d}} \end{array}$$

where *R*_*p*_, *R*_*d*_, and *C* are the lumped parameters of the model, representing the proximal and distal hydraulic resistances and the vessel compliance, respectively. The expression for *P*(*t*) can be derived by knowing *Q*(*t*) and by solving Equation 2. The lumped parameters for each branch are estimated according to the following Equations (60):


(3) $$\begin{array}{l} \begin{array}{c} R_{p_{i}}=\bar{R_{p}}\frac{A_{\text{tot}}}{A_{i}}\\ R_{d_{i}}=\bar{R_{d}}\frac{A_{\text{tot}}}{A_{i}}\;\text{for }i=1,\ldots ,n_{\text{outlets}}\\ C_{i}=\bar{C}\frac{A_{i}}{A_{\text{tot}}} \end{array} \end{array}$$

In Equation 3, *A*_*i*_ is the area of all the outlets (*A*_*i*_ = *A*_*BCA*_, *A*_*LCC*_, *A*_*LSUB*_, *A*_*Desc*_), and *A*_*tot*_ is the sum of all the areas of the outlets. The ${\bar{R}}_{p}$, ${\bar{R}}_{d}$, and $\bar{C}$ are the *overall* lumped parameters of the 3WKM model obtained from the systemic pressure estimation. Concerning the vessel walls, these were assumed to be impermeable and a no-slip condition was used for all the simulations.

Regarding the FSI structural boundary conditions, the *Desc* section was constrained with a fixed condition. The supra-aortic and aortic valve sections were constrained by defining a cylindrical reference system for each outlet and by constraining longitudinal and circumferential displacements.

### 2.4. WSS Analysis

All the post-processing analyses carried out to extract the WSS-based descriptors focused on the luminal wall of the ascending portion of the aorta. The TAWSS was also calculated for all the investigated cases. The TAWSS magnitude was measured in the last cardiac cycles by integrating each nodal WSS magnitude over the cardiac cycle as:


(4) $$\begin{array}{l} TAWSS=\frac{1}{T}\int_{0}^{T}\text{WSS}\left(t\right)dt \end{array}$$

## 3. Results

### 3.1. Smoothing

**Figure 4:**
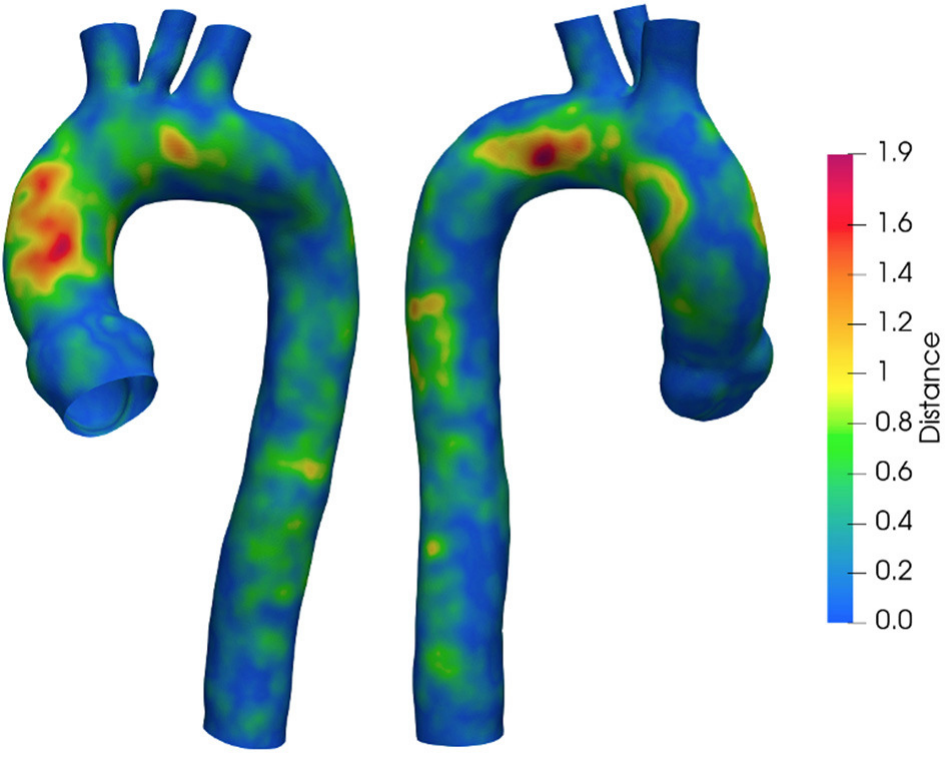
Geodesic distance between geometries with *S*_*L*_ and *S*_*H*_. Values are in mm.

To quantify the implemented smoothing range, the distances between the geometries obtained at different smoothing levels are reported. Figure 4 depicts the surface distance in the worst configuration: between the geometries with the lowest and highest smoothing level. The geodesic distance was carried out according to Dijkstra algorithm (61), implemented in the VMTK toolkit. Areas at maximum distances are observed as a consequence of calcium artifacts removal in the localized region of the vessel.

**Figure 5:**
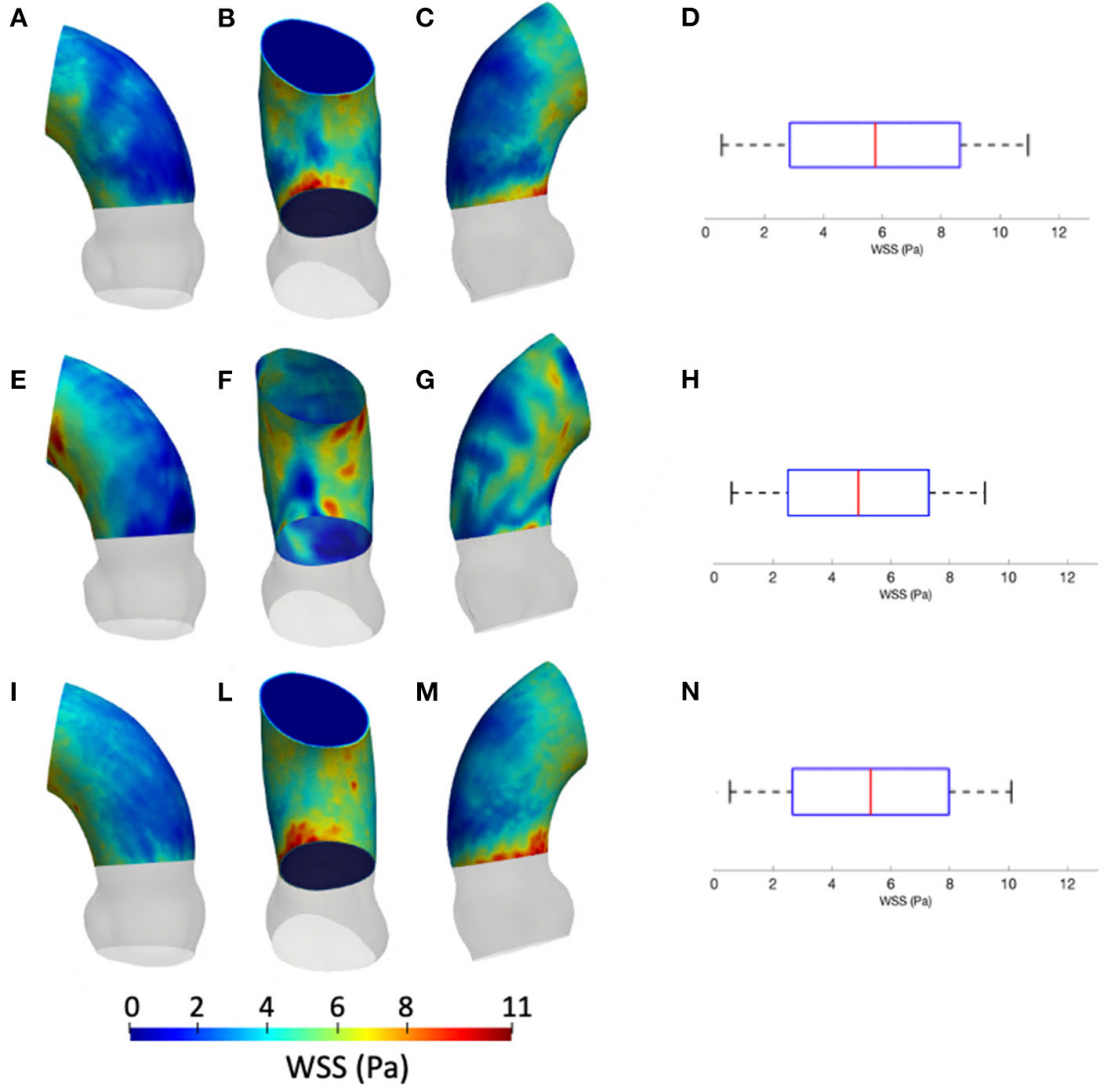
Contour map of the wall shear stress (WSS) at systolic peak for geometry at phase *p*_0_ considering the three different levels of smoothing: *S*_*L*_ [min: 0.5 Pa, avg: 5.9 Pa, max: 11 Pa] **(A–C)**, *S*_*M*_ [min: 0.4 Pa, avg: 4.9 Pa, max: 9.5 Pa] **(E–G)** and *S*_*H*_ [min: 0.4 Pa, avg: 5.1 Pa, max: 10 Pa] **(I–M)**. The corresponding box plots are reported in **D,H**, and **N**, respectively.

**Figure 6:**
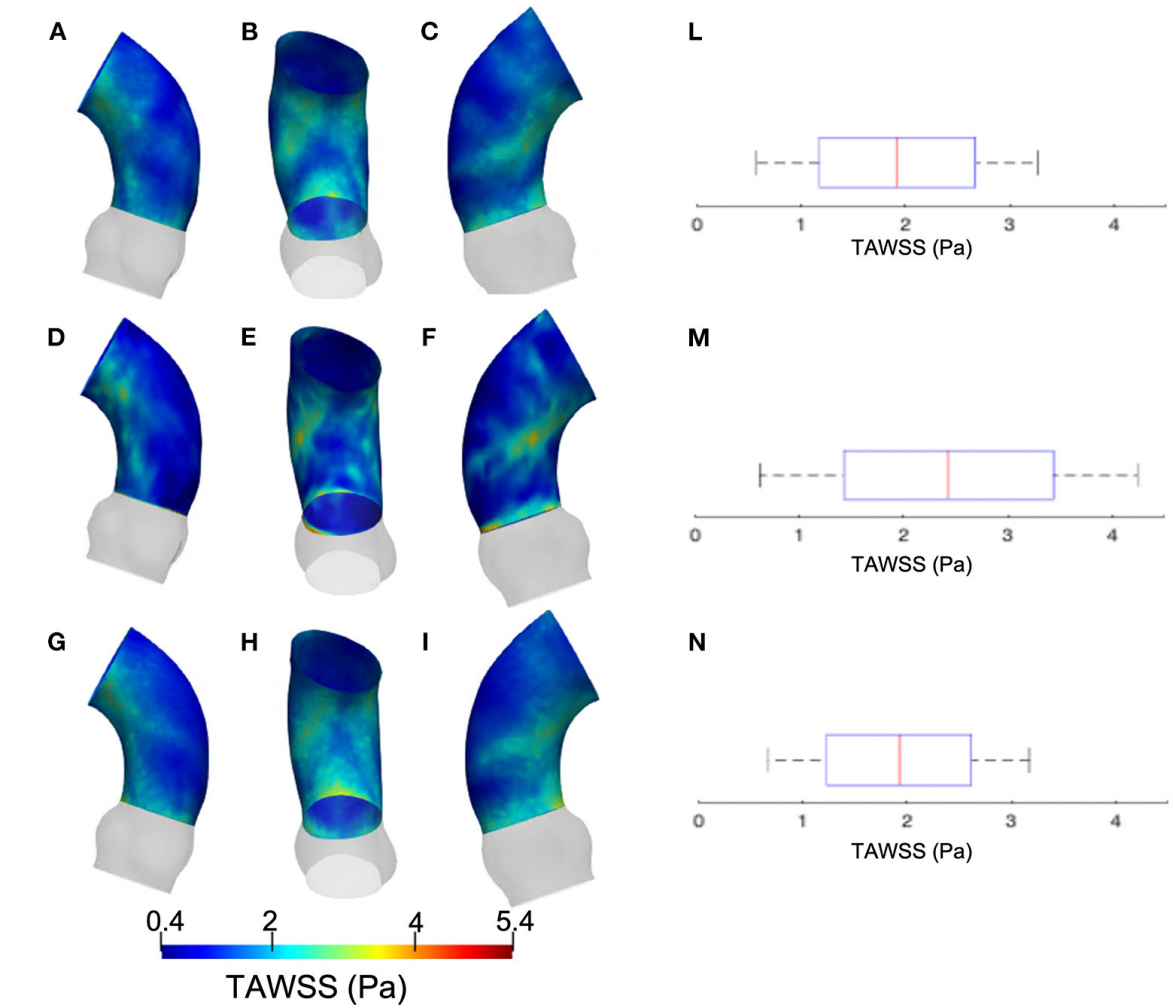
Contour map of the time-averaged WSS (TAWSS) for geometry at phase *p*_0_ considering three different level of smoothing: *S*_*L*_ [min: 0.4 Pa, avg: 1.6 Pa, max: 2.3 Pa] **(A–C)**, *S*_*M*_ [min: 0.4 Pa, avg: 2.0 Pa, max: 3.7 Pa] **(D–F)** and *S*_*H*_ [min: 0.4 Pa, avg: 2.0 Pa, max: 3.7 Pa] **(G–I)**. Box plot of the TAWSS are reported for *S*_*L*_ **(L)**, *S*_*M*_ **(M)**, and *S*_*H*_ **(N)**.

Figure 5 summarizes the WSS maps at the systolic peak for the aortic case with the three different levels of smoothing. The comparison in terms of CFD simulations was assessed for the phase *p*_0_ in the ascending aorta region. The box plots of Figures 5D,H,N describe the WSS element distribution in the area of interest for the three cases. The corresponding TAWSS analysis is instead reported in Figure 6. For both the systolic WSS and the TAWSS parameters, the reported range was comparable regardless of the smoothing level adopted, with peaks of 11 Pa and 5.4 Pa, respectively.

### 3.2. Boundary Conditions

Concerning the boundary conditions, the *R*_*p*_, *R*_*d*_, and the *C* computed according to 3WKM total, and to 3WKM tuning approaches for each outlet are reported in Table 1.

**Table 1:** Three-elements Windkessel models (3WKM) values: *R*_*p*_ and *R*_*d*_ are expressed in *Kg cm*^−4^ *s*^−1^ and *C* in *Kg*^−1^ *cm*^4^ *s*^2^.

	**BCA**	**LCC**	**LSUB**	**Desc**
*C*	2.18e−9	7.74e−10	1.56e−9	5.84e−9
*R_*p*_*	4.34e+7	1.22e+7	6.08e+7	1.62e+7
*R_*d*_*	6.80e+8	1.91e+9	9.52e+8	2.53e+8

### 3.3. CFD-Effect of Segmentation at Different Phases *p*_0_ and *p*_4_

**Figure 7:**
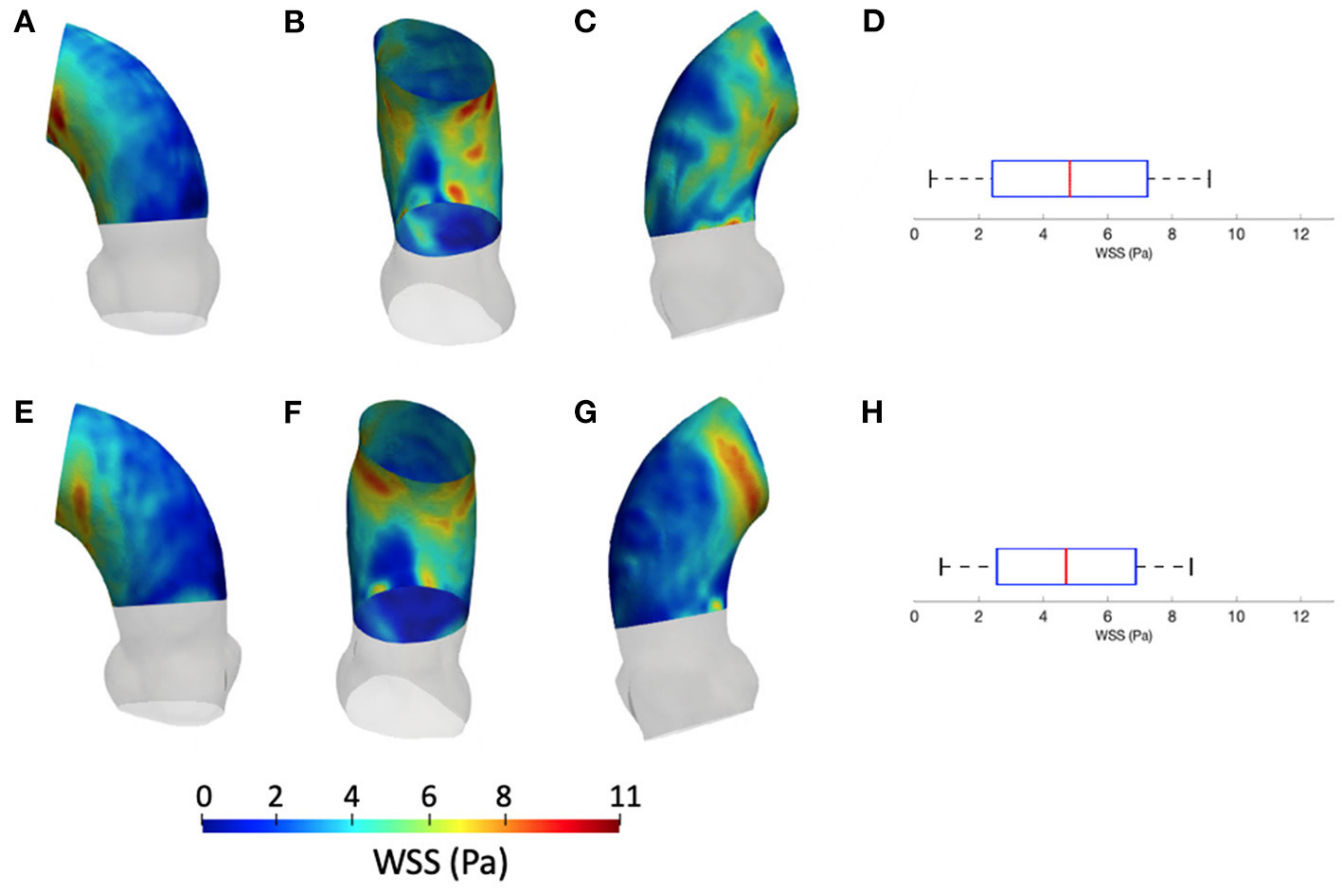
Contour map of the WSS at *p*_0_ [min: 0.4 Pa, avg: 4.9 Pa, max: 9.5 Pa] **(A–C)** and *p*_4_ [min: 0.6 Pa, avg: 4.5 Pa, max: 8.6 Pa] **(E–G)**. Box plots of the WSS at both configurations **(D,H)**.

**Figure 8:**
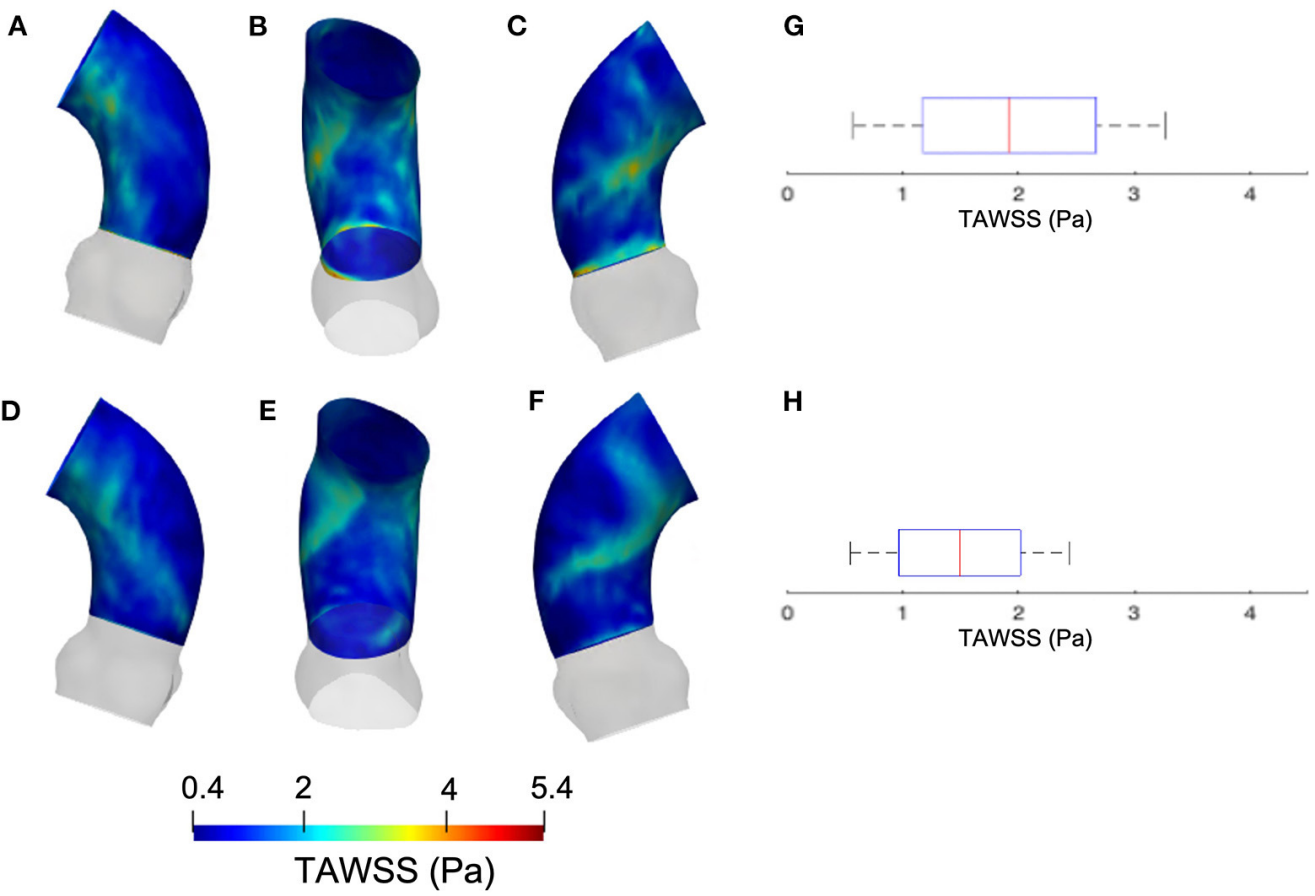
Contour map of the TAWSS at *p*_0_ [min: 0.4 Pa, avg: 2.3 Pa, max: 3.6 Pa] **(A–C)** and *p*_4_ [min: 0.3 Pa, avg: 1.2 Pa, max: 2.5 Pa] **(D–F)**. Box plots of the TAWSS are reported for *p*_0_ **(G)** and *p*_4_ **(H)**.

The results from the CFD at *p*_4_ are now compared with those of CFD at *p*_0_ with the *S*_*M*_ smoothing level. The contour maps from the *p*_4_ geometry are, instead, reported in terms of systolic WSS and TAWSS in Figures 7, 8, respectively. The box plots of peak systolic WSS are represented in Figures 7D,H. For the sake of comparison, the results of WSS and TAWSS at *p*_0_ with the *S*_*M*_ smoothing level were re-included in both figures.

### 3.4. Numerical Methods-CFD, CFD_*RBF*_, and FSI

**Figure 9:**
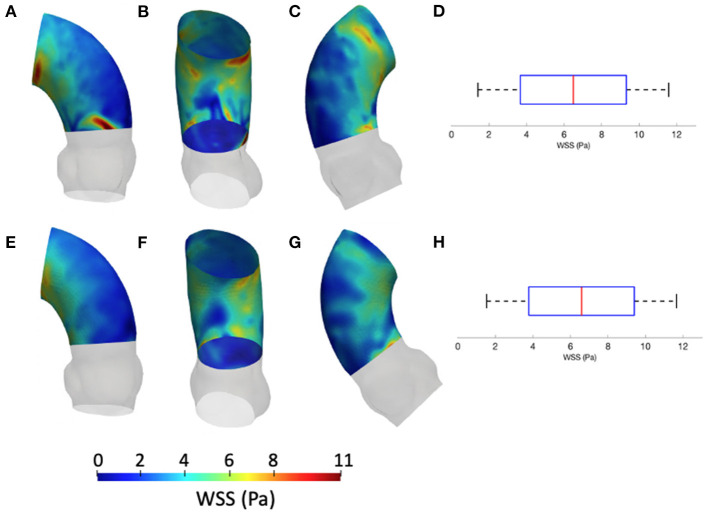
Contour map of the WSS for *CFD*_*RBF*_ [min: 1.1 Pa, avg: 6.6 Pa, max: 11.5 Pa] **(A–C)** and FSI [min: 1.3 Pa, avg: 6.5 Pa, max: 11.6 Pa] **(E–G)** simulations. Box plots of the WSS are reported for *CFD*_*RBF*_ **(D)** and FSI **(H)**.

**Figure 10:**
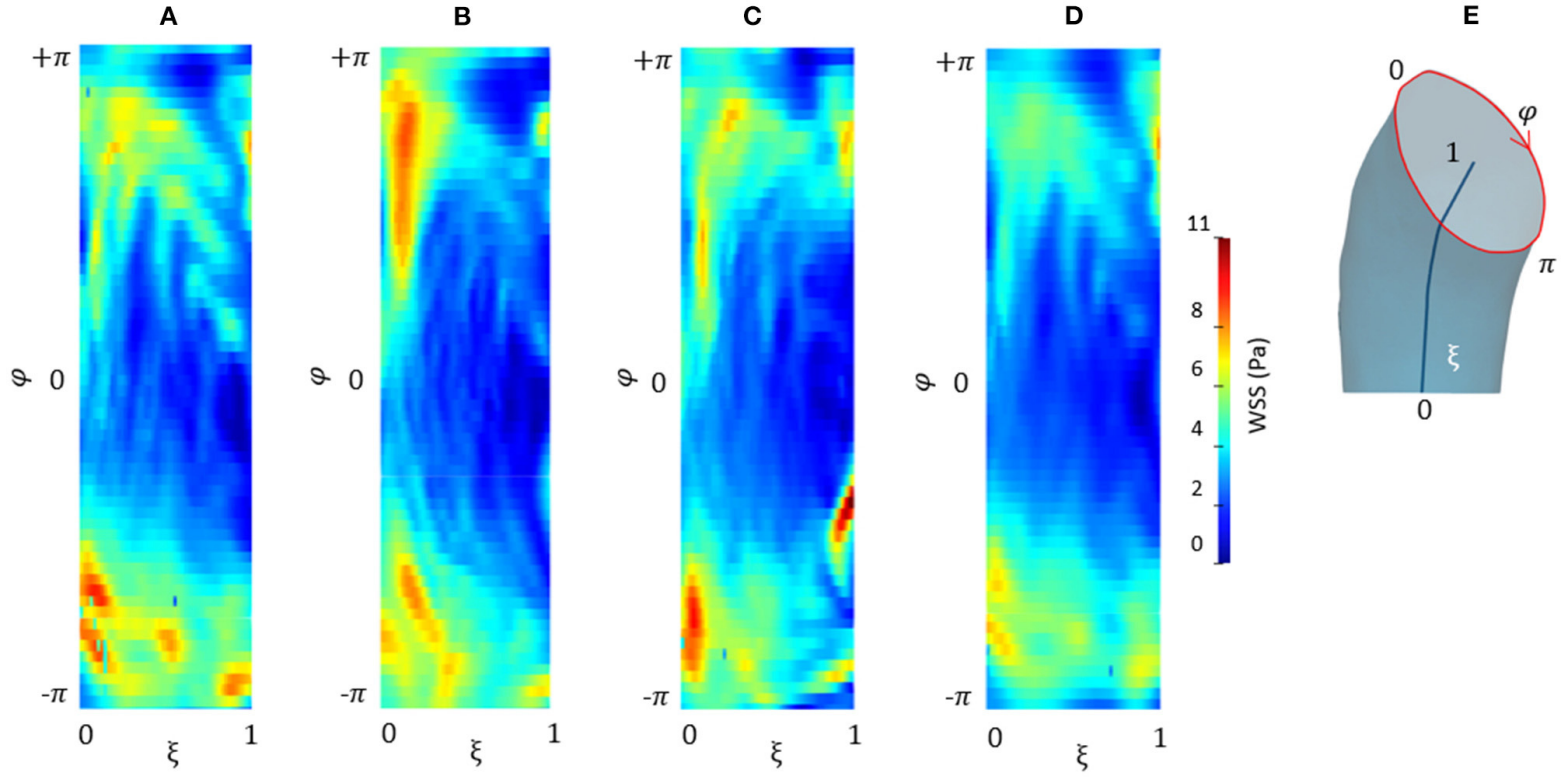
Contour map of the unfolded WSS for CFD at *p*_0_ **(A)**, CFD at *p*_4_ **(B)**, CFD_*RBF*_ **(C)**, and FSI **(D)** simulations. Circumferential and longitudinal coordinates are represented in **(E)**.

Figure 9 depicts the different maps in terms of WSS at systolic peak for the CFD_*RBF*_ and FSI simulations. The corresponding box plots for the distributions are depicted in Figures 9D,H. Additionally, for the sake of comparison, the maps of the unfolded surface of the ascending aorta surface are presented in Figure 10, for the CFD at both segmentation phases, the CFD_*RBF*_ and FSI cases.

## 4. Discussion

The effects of geometry variation on hemodynamics are assessed in a few studies. Analyses suggest that accurate lumen segmentation remains a challenging task in computational hemodynamic studies. The complexity of artifacts still affect the reconstruction algorithms of imaging techniques such as MRI (62). The groups of Abraham et al. (63) and Berthier et al. (64) investigated the effect of geometry variations on coronary vessels, while at the best of our knowledge, only one group focused on aorta (56). Both concluded that a small variation in geometry has a considerable effect on the predicted hemodynamics.

In the current study, different aspects of the modeling approaches for the hemodynamic analysis of *in silico* aortic geometries are presented. Starting from a patient-specific case, the different phases of processing and their effect on the analysis were assessed.

### 4.1. Surface Smoothing Effect

As a first step, the effect of surface smoothing after segmentation was defined. The distances between the *S*_*L*_ and *S*_*H*_ geometries are reported in Figure 4. The results of Figure 4 highlighted and quantified the effect of the implemented smoothing strategy, with maximum distances of 1.9 mm. It is worth stressing that the maximum values occurred in regions that required a local correction due to the presence of calcium artifacts. On the other hand, the global smoothing effects produced geodesic maximum distance differences less than 1 mm.

The results from Figures 5, 6 report the effect of the smoothing process in terms of WSS and TAWSS at the levels *S*_*L*_, *S*_*M*_, and *S*_*H*_. Beyond the map distributions, the box plot representation from Figures 5D,H,N allows for a comparison. From the mechanobiological point of view, the WSS is a patho-physiological stimulus at the basis of extra-cellular matrix disruption and elastic fiber degeneration (65). A link between low/oscillatory WSS and localized lesions of the vascular tissue and early disruption of endothelial cells was proven (66, 67). For this reason, WSS can be considered as a numerical predictor for tissue damage/pathology. Although the shear stress range is maintained in all three cases of Figure 5, it appears clear that the *S*_*L*_ case produces higher and more dispersed WSS values. On the contrary, no significant differences appear in terms of WSS distribution between the *S*_*M*_ and *S*_*H*_ levels. It is interesting to observe that the WSS trend changed as the calcification artifact removal was imposed. The presence of thrombi and calcifications are a common diseases affecting the abdominal district. The effect of calcifications presence was already underlined as an issue to be resolved to prepare a suitable geometry for numerical simulations (54). According to Ladich et al. (68), this pathology also exists in the thoracic aorta. In particular, both micro calcifications or extensive circumferential calcifications of the ascending aorta or aortic arch can be observed. While calcifications are associated with chronic systemic inflammatory diseases, their presence also affects the surface morphology and consequently the WSS. Similar results also were found for intracranial aneurysm (69) and more recently for the aorta in Perinajová et al. (56). In this last study, the importance of the smoothing applied in the segmentation step has been stressed out on CFD simulations. Given the WSS distribution stabilization after calcification removal, the level *S*_*M*_ was chosen as the minimum level of surface smoothing to allow a stable estimation of WSS and it was adopted for all the following numerical simulations. Additionally, it is worth noting that, while discrepancies in the local distribution exist, the higher values of stress were reported within the inner curvature of the ascending aorta for all levels *S*_*L*_, *S*_*M*_, and *S*_*H*_. These findings are important and give rise to reflection from a clinical perspective, for instance in patients affected by aortitis or porcelain aortas.

### 4.2. Segmentation Effect

Concerning the results of the CFD from the systolic segmented phase *p*_4_, the distribution of WSS and TAWSS can be analyzed from Figures 7, 8 and from the unfolded maps of Figures 10A,B. The box plot in Figures 7D,H displays results of WSS in accordance with the previous simulations segmented from phase *p*_0_. Nevertheless, the distribution from Figures 10A,B reveals a different mapping between the CFD at the two phases. This aspect underlines again the effect of geometry on the WSS behavior within the aortic domain.

### 4.3. Numerical Methods Effect

The implementation of numerical methods was investigated as well. Beyond the rigid wall CFD results, already presented, the CFD_*RBF*_ and FSI approach produced the WSS distributions of Figure 9. The box plots from Figures 9D,H allowed for distribution comparison. Except for the presence of peak areas, it appears that the distribution presents the same average and range for both modalities. No significant difference emerged from the comparison of distributions of Figures 9D,H. The same trend is confirmed by the unfolded maps of Figure 10, in which appears a difference in terms of WSS between both CFD_*RBF*_ and FSI approaches and the CFDs. The reported shear stress ranges appear to match, nevertheless, the CFD_*RBF*_ produced peaks, especially in the inner curvature zone of the ascending aorta. As an overall consideration, the investigation of the CFDs box plot in Figures 7, 9 shows the differences arising from the wall motion assumption. From Figure 10, it appears that the introduction of wall motion within the model produced a shift toward higher WSS values, regardless of the method used to implement it. This aspect corroborates the necessity to include a realistic wall motion model within the simulation of the aorta, as it significantly affects the result. Comparisons in terms of rigid and moving wall numerical simulations for the aorta were reported already in the state of the art (46). The presented results highlighted the presence of a WSS difference according to the numerical technique adopted, however, the discrepancy is expected to diminish as long as the tissue is rigid. An additional point to underline is that the FSI approach requires the estimation step of material properties. While the FSI and CFD_*RBF*_ manifested no significant difference in terms of WSS distribution, the CFD_*RBF*_ method did not require any hypothesis on the material constitutive model. For this reason, no strong assumption and indirect *in vivo* measures was introduced (27, 28). The CFD_*RBF*_ method was computed exclusively on the basis of the segmentation of the cardiac phases. Consequently, the CFD_*RBF*_ approach presented the advantage of an a-priori exclusion of the intrinsic uncertainties given by material characterization and modeling, which were demonstrated to be responsible for inaccuracies (70, 71). Recent studies proposed this approach to study the effect of aortic flow by imposing valve kinematics (72, 73).

The current study presents several points of development for the future. The effects of smoothing levels and different phase segmentation were carried out on a single patient-specific case. Nevertheless, the analyses of WSS distributions produced differences in terms of map distribution for given levels of smoothness and confirmed the influence of geometrical/morphological factors on the WSS, while the less evident map distribution differences were found for TAWSS.

The choice of different image phases for reconstruction implied differences in hemodynamic results. This demonstrates that the sources of morphological uncertainties are not only the segmentation technique and smoothing approach but also the different possible geometries that can be defined for the same patient-specific case. As the number of cases recruited is increased, a similar trend is expected. An additional point of development for the future is to adopt the CFD_*RBF*_ approach to account for the aortic root movement. It was demonstrated that the aortic root and left ventricle kinematics might have an influence on the numerical modeling of the aorta (74). The CFD_*RBF*_ method was demonstrated to be a potential tool to integrate the patient-specific displacement-based imaging data with numerical simulations. For this reason, it appears as a suitable way to include rigid motions like the ones occurring in the ventricle and aortic root. The results presented within the current study were focused on computational methods. Nevertheless, an interesting point of development would be the inclusion of experimental methods of validation like the adoption of mock circulatory loops (75, 76). In the *in silico* scenario, optical techniques like particle image velocimetry (77) could be adopted for velocity profiles comparison.

## 5. Conclusion

In summary, with the presented manuscript, different sources of possible uncertainties within the analysis of aorta hemodynamics were reviewed and assessed. It was successfully demonstrated that image processing approaches to influence the results in terms of shear stress. Additionally, the importance of introducing wall motion was assessed and its effects on the WSS spatial distribution were confirmed. In conclusion, with the current study, an overview of the effect of given approaches on the analysis of aorta hemodynamics was given, with the objective of accounting for possible inaccuracies to improve the faithfulness of the *in-silico* models.

## Data Availability Statement

The original contributions presented in the study are included in the article, further inquiries can be directed to the corresponding author.

## Ethics Statement

The studies involving human participants were reviewed and approved by Fondazione Toscana G. Monasterio internal local committee. The patients/participants provided their written informed consent to participate in this study.

## Author Contributions

SC, KC, EV, and EG: formal analysis and methodology. KC, EV, and EG: investigation. KC and EV: visualization. SC and EV: writing—original draft and writing—review and editing. SC: conceptualization, supervision and project administration, and resources. All authors contributed to the article and approved the submitted version.

## Funding

This project was funded from the Grant Number 936584 - IPR - 2018 in response to Tender JRC/IPR/2018/F3/0035/OC Reviews on Non-animal Methods in Use for Biomedical Research divided in 5 lots. Lot 1: Cardiovascular diseases.

## Conflict of Interest

The authors declare that the research was conducted in the absence of any commercial or financial relationships that could be construed as a potential conflict of interest. The handling editor declared a past collaboration with two of the authors SC and EG.

## Publisher's Note

All claims expressed in this article are solely those of the authors and do not necessarily represent those of their affiliated organizations, or those of the publisher, the editors and the reviewers. Any product that may be evaluated in this article, or claim that may be made by its manufacturer, is not guaranteed or endorsed by the publisher.

## References

[1] Xiong G Sun P Zhou H Ha S ó Hartaigh B Truong QA. Comprehensive modeling and visualization of cardiac anatomy and physiology from CT imaging and computer simulations. IEEE Trans Vis Comput Graph. (2016) 23:1014–28. 10.1109/TVCG.2016.2520946 26863663 PMC4975682

[2] Shang EK Lai E Pouch AM Hinmon R Gorman RC Gorman JH III. Validation of semiautomated and locally resolved aortic wall thickness measurements from computed tomography. J Vasc Surg. (2015) 61:1034–40. 10.1016/j.jvs.2013.11.065 24388698 PMC4121383

[3] Hino T Kamitani T Sagiyama K Yamasaki Y Matsuura Y Tsutsui S. Detectability of the artery of Adamkiewicz on computed tomography angiography of the aorta by using ultra-high-resolution computed tomography. Jpn J Radiol. (2020) 38:658–65. 10.1007/s11604-020-00943-3 32170567

[4] Amsallem M Ou P Milleron O Henry-Feugeas MC Detaint D Arnoult F. Comparative assessment of ascending aortic aneurysms in Marfan patients using ECG-gated computerized tomographic angiography versus trans-thoracic echocardiography. Int J Cardiol. (2015) 184:22–7. 10.1016/j.ijcard.2015.01.086 25705006

[5] Pepe A Li J Rolf-Pissarczyk M Gsaxner C Chen X Holzapfel GA. Detection, segmentation, simulation and visualization of aortic dissections: a review. Med Image Anal. (2020) 65:101773. 10.1016/j.media.2020.101773 32738647

[6] Boufi M Guivier-Curien C Loundou A Deplano V Boiron O Chaumoitre K. Morphological analysis of healthy aortic arch. Eur J Vasc Endovasc Surg. (2017) 53:663–670. 10.1016/j.ejvs.2017.02.023 28351602

[7] Shang EK Nathan DP Fairman RM Bavaria JE Gorman RC Gorman JH III. Use of computational fluid dynamics studies in predicting aneurysmal degeneration of acute type B aortic dissections. J Vasc Surg. (2015) 62:279–84. 10.1016/j.jvs.2015.02.048 25935270 PMC6685432

[8] Parodi J Berguer R Carrascosa P Khanafer K Capunay C Wizauer E. Sources of error in the measurement of aortic diameter in computed tomography scans. J Vasc Surg. (2014) 59:74–9. 10.1016/j.jvs.2013.07.005 23958070

[9] Saeed M Van TA Krug R Hetts SW Wilson MW. Cardiac MR imaging: current status and future direction. Cardiovasc Diagn Ther. (2015) 5:290. 10.3978/j.issn.2223-3652.2015.06.07 26331113 PMC4536478

[10] Ohyama Y Redheuil A Kachenoura N Ambale Venkatesh B Lima JA. Imaging insights on the aorta in aging. Circ Cardiovasc Imaging. (2018) 11:e005617. 10.1161/CIRCIMAGING.117.005617 29653929 PMC6029255

[11] Gabbour M Schnell S Jarvis K Robinson JD Markl M Rigsby CK. 4-D flow magnetic resonance imaging: blood flow quantification compared to 2-D phase-contrast magnetic resonance imaging and Doppler echocardiography. Pediatric Radiol. (2015) 45:804–13. 10.1007/s00247-014-3246-z 25487721 PMC4450116

[12] Burris NS Hope MD. 4D flow MRI applications for aortic disease. Magn Reson Imaging Clin. (2015) 23:15–23. 10.1016/j.mric.2014.08.006 25476670 PMC5127263

[13] Pewowaruk R Roldán-Alzate A. 4D flow MRI estimation of boundary conditions for patient specific cardiovascular simulation. Ann Biomed Eng. (2019) 47:1786–98. 10.1007/s10439-019-02285-2 31069584 PMC7328374

[14] Miyazaki S Itatani K Furusawa T Nishino T Sugiyama M Takehara Y. Validation of numerical simulation methods in aortic arch using 4D Flow MRI. Heart Vessels. (2017) 32:1032–44. 10.1007/s00380-017-0979-2 28444501 PMC5519664

[15] Friman O Hennemuth A Harloff A Bock J Markl M Peitgen HO. Probabilistic 4D blood flow tracking and uncertainty estimation. Med Image Anal. (2011) 15:720–8. 10.1016/j.media.2011.06.002 21719342

[16] Hess AT Bissell MM Ntusi NA Lewis AJ Tunnicliffe EM Greiser A. Aortic 4D flow: Quantification of signal-to-noise ratio as a function of field strength and contrast enhancement for 1.5 T, 3T, and 7T. Magn Reson Med. (2015) 73:1864–71. 10.1002/mrm.25317 24934930

[17] Soudah E Casacuberta J Gamez-Montero PJ Perez J Rodriguez-Cancio M Raush G. Estimation of wall shear stress using 4D flow cardiovascular MRI and computational fluid dynamics. J Mech Med Biol. (2017) 17:1750046. 10.1142/S0219519417500464

[18] van Ooij P Potters WV Collins J Carr M Carr J Malaisrie SC. Characterization of abnormal wall shear stress using 4D flow MRI in human bicuspid aortopathy. Ann Biomed Eng. (2015) 43:1385–97. 10.1007/s10439-014-1092-7 25118671 PMC4329118

[19] Manchester EL Pirola S Salmasi MY O'Regan DP Athanasiou T Xu XY. Analysis of turbulence effects in a patient-specific aorta with aortic valve stenosis. Cardiovasc Eng Technol. (2021) 12:438–53. 10.1007/s13239-021-00536-9 33829405 PMC8354935

[20] Bozzi S Morbiducci U Gallo D Ponzini R Rizzo G Bignardi C. Uncertainty propagation of phase contrast-MRI derived inlet boundary conditions in computational hemodynamics models of thoracic aorta. Comput Methods Biomech Biomed Engin. (2017) 20:1104–112. 10.1080/10255842.2017.1334770 28553722

[21] Vivoli G Gasparotti E Rezzaghi M Cerone E Mariani M Landini L. Simultaneous functional and morphological assessment of left atrial appendage by 3D virtual models. J Healthc Eng. (2019) 2019:7095845. 10.1155/2019/7095845 31249656 PMC6556349

[22] Brüning J Hellmeier F Yevtushenko P Kühne T Goubergrits L. Uncertainty quantification for non-invasive assessment of pressure drop across a coarctation of the aorta using CFD. Cardiovasc Eng Technol. (2018) 9:582–96. 10.1007/s13239-018-00381-3 30284186

[23] Finnegan R Dowling J Koh ES Tang S Otton J Delaney G. Feasibility of multi-atlas cardiac segmentation from thoracic planning CT in a probabilistic framework. Phys Med Biol. (2019) 64:085006. 10.1088/1361-6560/ab0ea6 30856618

[24] Litjens G Kooi T Bejnordi BE Setio AAA Ciompi F Ghafoorian M. A survey on deep learning in medical image analysis. Med Image Anal. (2017) 42:60–88. 10.1016/j.media.2017.07.005 28778026

[25] Maher G Parker D Wilson N Marsden A. Neural network vessel lumen regression for automated lumen cross-section segmentation in cardiovascular image-based modeling. Cardiovasc Eng Technol. (2020) 11:621–35. 10.1007/s13239-020-00497-5 33179176 PMC7785699

[26] Hwang M Yoo J Kim H Hwang C Mackay K Hemstreet O. Validity and reliability of aortic pulse wave velocity and augmentation index determined by the new cuff-based SphygmoCor Xcel. J Hum Hypertens. (2014) 28:475–81. 10.1038/jhh.2013.144 24430704

[27] Fanni B Sauvage E Celi S Norman W Vignali E Landini L. A proof of concept of a non-invasive image-based material characterization method for enhanced patient-specific computational modeling. Cardiovasc Eng Technol. (2020) 11:532–43. 10.1007/s13239-020-00479-7 32748364

[28] Farzaneh S Trabelsi O Avril S. Inverse identification of local stiffness across ascending thoracic aortic aneurysms. Biomech Model Mechanobiol. (2019) 18:137–53. 10.1007/s10237-018-1073-0 30145618

[29] Bosi GM Capelli C Khambadkone S Taylor AM Schievano S. Patient-specific finite element models to support clinical decisions: a lesson learnt from a case study of percutaneous pulmonary valve implantation. Catheter Cardiovasc Interv. (2015) 86:1120–30. 10.1002/ccd.25944 25855063

[30] Bosi GM Biffi B Biglino G Lintas V Jones R Tzamtzis S. Can finite element models of ballooning procedures yield mechanical response of the cardiovascular site to overexpansion? J Biomech. (2016) 49:2778–84. 10.1016/j.jbiomech.2016.06.021 27395759 PMC5522534

[31] Duprey A Trabelsi O Vola M Favre JP Avril S. Biaxial rupture properties of ascending thoracic aortic aneurysms. Acta Biomater. (2016) 42:273–85. 10.1016/j.actbio.2016.06.028 27345137

[32] Vignali E di Bartolo F Gasparotti E Malacarne A Concistré G Chiaramonti F. Correlation between micro and macrostructural biaxial behavior of ascending thoracic aneurysm: a novel experimental technique. Med Eng Phys. (2020) 86:78–85. 10.1016/j.medengphy.2020.10.012 33261737

[33] Vignali E Gasparotti E Landini L Celi S. Development and realization of an experimental bench test for synchronized small angle light scattering and biaxial traction analysis of tissues. Electronics. (2021) 10:386. 10.3390/electronics10040386

[34] Vignali E Gasparotti E Capellini K Fanni BM Landini L Positano V. Modeling biomechanical interaction between soft tissue and soft robotic instruments: importance of constitutive anisotropic hyperelastic formulations. Int J Rob Res. (2021) 40:224–35. 10.1177/0278364920927476

[35] Ferrara A Morganti S Totaro P Mazzola A Auricchio F. Human dilated ascending aorta: mechanical characterization via uniaxial tensile tests. J Mech Behav Biomed Mater. (2016) 53:257–71. 10.1016/j.jmbbm.2015.08.021 26356765

[36] Schroeder F Polzer S Slažanskỳ M Man V Skácel P. Predictive capabilities of various constitutive models for arterial tissue. J Mech Behav Biomed Mater. (2018) 78:369–80. 10.1016/j.jmbbm.2017.11.035 29220821

[37] Cosentino F Agnese V Raffa GM Gentile G Bellavia D Zingales M. On the role of material properties in ascending thoracic aortic aneurysms. Comput Biol Med. (2019) 109:70–8. 10.1016/j.compbiomed.2019.04.022 31035073

[38] Boccadifuoco A Mariotti A Capellini K Celi S Salvetti MV. Validation of numerical simulations of thoracic aorta hemodynamics: comparison with in vivo measurements and stochastic sensitivity analysis. Cardiovasc Eng Technol. (2018) 9:688–706. 10.1007/s13239-018-00387-x 30357714

[39] Vignali E Gasparotti E Celi S Avril S. Fully-coupled FSI computational analyses in the ascending thoracic aorta using patient-specific conditions and anisotropic material properties. Front Physiol. (2021) 12:732561. 10.3389/fphys.2021.732561 34744774 PMC8564074

[40] Pons R Guala A Rodríguez-Palomares JF Cajas J Dux-Santoy L Teixidó-Tura G. Fluid-structure interaction simulations outperform computational fluid dynamics in the description of thoracic aorta haemodynamics and in the differentiation of progressive dilation in Marfan syndrome patients. R Soc Open Sci. (2020) 7:191752. 10.1098/rsos.191752 32257331 PMC7062053

[41] Celi S Berti S. Three-dimensional sensitivity assessment of thoracic aortic aneurysm wall stress: a probabilistic finite-element study. Eur J Cardiothor Surg. (2014) 45:467–75. 10.1093/ejcts/ezt400 23921161

[42] Iliopoulos DC Kritharis EP Giagini AT Papadodima SA Sokolis DP. Ascending thoracic aortic aneurysms are associated with compositional remodeling and vessel stiffening but not weakening in age-matched subjects. J Thorac Cardiovasc Surg. (2009) 137:101–9. 10.1016/j.jtcvs.2008.07.023 19154911

[43] Pirola S Jarral O O'Regan D Asimakopoulos G Anderson J Pepper J. Computational study of aortic hemodynamics for patients with an abnormal aortic valve: the importance of secondary flow at the ascending aorta inlet. APL Bioeng. (2018) 2:026101. 10.1063/1.5011960 31069298 PMC6481743

[44] Youssefi P Gomez A Arthurs C Sharma R Jahangiri M Alberto Figueroa C. Impact of patient-specific inflow velocity profile on hemodynamics of the thoracic aorta. J Biomech Eng. (2018) 140:1–14. 10.1115/1.4037857 28890987

[45] Campobasso R Condemi F Viallon M Croisille P Campisi S Avril S. Evaluation of peak wall stress in an ascending thoracic aortic aneurysm using FSI simulations: effects of aortic stiffness and peripheral resistance. Cardiovasc Eng Technol. (2018) 9:707–22. 10.1007/s13239-018-00385-z 30341731

[46] Mendez V Di Giuseppe M Pasta S. Comparison of hemodynamic and structural indices of ascending thoracic aortic aneurysm as predicted by 2-way FSI, CFD rigid wall simulation and patient-specific displacement-based FEA. Comput Biol Med. (2018) 100:221–9. 10.1016/j.compbiomed.2018.07.013 30053678

[47] Capellini K Vignali E Costa E Gasparotti E Biancolini ME Landini L. Computational fluid dynamic study for aTAA hemodynamics: an integrated image-based and radial basis functions mesh morphing approach. J Biomech Eng. (2018) 140:111007. 10.1115/1.4040940 30098137

[48] Capellini K Gasparotti E Cella U Costa E Fanni BM Groth C. A novel formulation for the study of the ascending aortic fluid dynamics with in vivo data. Med Eng Phys. (2021) 91:68–78. 10.1016/j.medengphy.2020.09.005 33008714

[49] Biancolini ME Capellini K Costa E Groth C Celi S. Fast interactive CFD evaluation of hemodynamics assisted by RBF mesh morphing and reduced order models: the case of aTAA modelling. Int J Interact Design Manufact. (2020) 14:1227–38. 10.1007/s12008-020-00694-5

[50] Boccadifuoco A Mariotti A Celi S Martini N Salvetti MV. Effects of inlet conditions in the simulation of hemodynamics in a thoracic aortic aneurysm. In: Proceedings of the 23rd Conference of the Italian Association of Theoretical and Applied Mechanics (AIMETA 2017). Vol. 2. Salerno (2017). p. 1706-1724.

[51] Antonuccio MN Mariotti A Celi S Salvetti MV. Effects of the distribution in space of the velocity-inlet condition in hemodynamic simulations of the thoracic aorta. In: Bioinformatics and Biomedical Engineering: 8th International Work-Conference, IWBBIO. Granada, May 6–8 (2020). p. 63.

[52] Mariotti A Celi S Salvetti M. Hemodynamics and stresses in numerical simulations of the thoracic aorta - Part I: stochastic sensitivity analysis to inlet flow-rate waveform. Comput Fluids. (2021) 9. 10.1016/j.compfluid.2021.10512330357714

[53] Bade R Haase J Preim B. Comparison of fundamental mesh smoothing algorithms for medical surface models. In: SimVis. vol. 6. Citeseer (2006). p. 289–304.

[54] Salman HE Ramazanli B Yavuz MM Yalcin HC. Biomechanical investigation of disturbed hemodynamics-induced tissue degeneration in abdominal aortic aneurysms using computational and experimental techniques. Front Bioeng Biotechnol. (2019) 7:111. 10.3389/fbioe.2019.00111 31214581 PMC6555197

[55] Taubin G. Curve and surface smoothing without shrinkage. In: Proceedings of IEEE International Conference on Computer Vision. Cambridge, MA: IEEE (1995). p. 852–7.

[56] Perinajová R Juffermans JF Westenberg JJ van der Palen RL van den Boogaard PJ Lamb HJ. Geometrically induced wall shear stress variability in CFD-MRI coupled simulations of blood flow in the thoracic aortas. Comput Biol Med. (2021) 133:104385. 10.1016/j.compbiomed.2021.104385 33894502

[57] Armour CH Guo B Pirola S Saitta S Liu Y Dong Z. The influence of inlet velocity profile on predicted flow in type B aortic dissection. Biomech Model Mechanobiol. (2021) 20:481–90. 10.1007/s10237-020-01395-4 33068193 PMC7979630

[58] Caballero A Laín S. A review on computational fluid dynamics modelling in human thoracic aorta. Cardiovasc Eng Technol. (2013) 4:103–30. 10.1007/s13239-013-0146-631638698

[59] Degroote J Swillens A Bruggeman P Haelterman R Segers P Vierendeels J. Simulation of fluid-structure interaction with the interface artificial compressibility method. Int J Numer Method Biomed Eng. (2010) 26:276–89. 10.1002/cnm.127625855820

[60] Boccadifuoco A Mariotti A Celi S Martini N Salvetti M. Impact of uncertainties in outflow boundary conditions on the predictions of hemodynamic simulations of ascending thoracic aortic aneurysms. Comput Fluids. (2018) 165:96–115. 10.1016/j.compfluid.2018.01.012

[61] Dijkstra EW. A note on two problems in connexion with graphs. Numer Math. (1959) 1:269–71. 10.1007/BF01386390

[62] Jayendiran R Condemi F Campisi S Viallon M Croisille P Avril S. Computational prediction of hemodynamical and biomechanical alterations induced by aneurysm dilatation in patient-specific ascending thoracic aortas. Int J Numer Method Biomed Eng. (2020) 36:e3326. 10.1002/cnm.3326 32087044

[63] Abraham J Sparrow EM Lovik R. Unsteady, three-dimensional fluid mechanic analysis of blood flow in plaque-narrowed and plaque-freed arteries. Int J Heat Mass Transf . (2008) 51:5633–5641. 10.1016/j.ijheatmasstransfer.2008.04.03815117028

[64] Berthier B Bouzerar R Legallais C. Blood flow patterns in an anatomically realistic coronary vessel: influence of three different reconstruction methods. J Biomech. (2002) 35:1347–56. 10.1016/S0021-9290(02)00179-3 12231280

[65] Guzzardi DG Barker AJ Van Ooij P Malaisrie SC Puthumana JJ Belke DD. Valve-related hemodynamics mediate human bicuspid aortopathy: insights from wall shear stress mapping. J Am Coll Cardiol. (2015) 66:892–900. 10.1016/j.jacc.2015.06.1310 26293758 PMC4545965

[66] Peiffer V Sherwin SJ Weinberg PD. Does low and oscillatory wall shear stress correlate spatially with early atherosclerosis? A systematic review. Cardiovasc Res. (2013) 99:242–50. 10.1093/cvr/cvt044 23459102 PMC3695746

[67] Gallo D Bijari PB Morbiducci U Qiao Y Xie Y Etesami M. Segment-specific associations between local haemodynamic and imaging markers of early atherosclerosis at the carotid artery: an in vivo human study. J R Soc Interface. (2018) 15:20180352. 10.1098/rsif.2018.0352 30305419 PMC6228482

[68] Ladich E Yahagi K Romero ME Virmani R. Vascular diseases: aortitis, aortic aneurysms, and vascular calcification. Cardiovasc Pathol. (2016) 25:432–41. 10.1016/j.carpath.2016.07.002 27526100

[69] Valen-Sendstad K Bergersen AW Shimogonya Y Goubergrits L Bruening J Pallares J. Real-world variability in the prediction of intracranial aneurysm wall shear stress: the 2015 international aneurysm CFD challenge. Cardiovasc Eng Technol. (2018) 9:544–64. 10.1007/s13239-018-00374-2 30203115 PMC6290689

[70] Biehler J Gee MW Wall WA. Towards efficient uncertainty quantification in complex and large-scale biomechanical problems based on a Bayesian multi-fidelity scheme. Biomech Model Mechanobiol. (2015) 14:489–513. 10.1007/s10237-014-0618-0 25245816

[71] Viceconti M Juárez MA Curreli C Pennisi M Russo G Pappalardo F. Credibility of in silico trial technologies–A theoretical framing. IEEE J Biomed Health Inform. (2019) 24:4–13. 10.1109/JBHI.2019.2949888 31670687

[72] Geronzi L Gasparotti E Capellini K Cella U Groth C Porziani S. Advanced Radial Basis Functions mesh morphing for high fidelity Fluid-Structure Interaction with known movement of the walls: simulation of an aortic valve. In: International Conference on Computational Science. Amsterdam: Springer (2020). p. 280–93.

[73] Geronzi L Gasparotti E Capellini K Cella U Groth C Porziani S. High fidelity fluid-structure interaction by radial basis functions mesh adaption of moving walls: a workflow applied to an aortic valve. J Comput Sci. (2021) 51:101327. 10.1016/j.jocs.2021.101327

[74] Guala A Teixidó-Tura G Rodríguez-Palomares J Ruiz-Muñoz A Dux-Santoy L Villalva N. Proximal aorta longitudinal strain predicts aortic root dilation rate and aortic events in Marfan syndrome. Eur Heart J. (2019) 40:2047–2055. 10.1093/eurheartj/ehz191 30977783

[75] Mariotti A Vignali E Gasparotti E Capellini K Celi S Salvetti MV. Comparison between numerical and MRI data of ascending aorta hemodynamics in a circulatory mock loop. In: Conference of the Italian Association of Theoretical and Applied Mechanics. Rome: Springer (2019). p. 898–907. 10.1007/978-3-030-41057-5_73

[76] Vignali E Gasparotti E Fanni BM Ait-Ali L Positano V Landini L. Development of a fully controllable real-time pump to reproduce left ventricle physiological flow. In: Conference of the Italian Association of Theoretical and Applied Mechanics. Springer (2019). p. 908–19.

[77] Keshavarz-Motamed Z Garcia J Gaillard E Maftoon N Di Labbio G Cloutier G. Effect of coarctation of the aorta and bicuspid aortic valve on flow dynamics and turbulence in the aorta using particle image velocimetry. Exp Fluids. (2014) 55:1–16. 10.1007/s00348-014-1696-6

